# Intra- and Post-match Time-Course of Indicators Related to Perceived and Performance Fatigability and Recovery in Elite Youth Soccer Players

**DOI:** 10.3389/fphys.2019.01383

**Published:** 2019-11-15

**Authors:** Philipp Kunz, Christoph Zinner, Hans-Christer Holmberg, Billy Sperlich

**Affiliations:** ^1^Integrative & Experimental Exercise Science and Training, Institute of Sport Science, University of Würzburg, Würzburg, Germany; ^2^Department of Sport, University of Applied Sciences for Police and Administration of Hesse, Wiesbaden, Germany; ^3^Department of Health Sciences, Mid Sweden University, Östersund, Sweden; ^4^Department of Physiology and Pharmacology, Karolinska Institute, Stockholm, Sweden

**Keywords:** soccer (football), youth, match load, fatigue, intermittent exercise, performance

## Abstract

**Purpose:**

Our aims were to examine (i) the internal load during simulated soccer match-play by elite youth players; and (ii) the time-course of subsequent recovery from perceived and performance fatigability.

**Methods:**

Eleven male youth players (16 ± 1 years, 178 ± 7 cm, 67 ± 7 kg) participated in a 2 × 40-min simulated soccer match, completing 30 rounds (160 s each) with every round including multidirectional and linear sprinting (LS20m), jumping (CMJ) and running at different intensities. During each round, LS20m, CMJ, agility, heart rate (HR), oxygen uptake (VO2), energy expenditure (EE), substrate utilization and perceived exertion RPE were assessed. In addition, the blood level of lactate (Lac) was obtained after each of the five rounds. Creatine kinase (CK) concentration, maximal voluntary isometric knee extension and flexion, CMJ, number of skippings in 30 s, and subjective ratings on the Acute Recovery and Stress Scale (ARSS) were examined before and immediately, 24 and 48 h after the simulation.

**Results:**

During the game %HR_peak_ (*p* < 0.05, *d* = 1.08), %VO_2__peak_ (*p* < 0.05; *d* = 0.68), Lac (*p* < 0.05, *d* = 2.59), RPE_total_ (*p* < 0.05, *d* = 4.59), and RPE_legs_ (*p* < 0.05, *d* = 4.45) all increased with time during both halves (all *p* < 0.05). Agility improved (*p* < 0.05, *d* = 0.70) over the time-course of the game, with no changes in LS20m (*p* ≥ 0.05, *d* = 0.34) or CMJ (*p* ≥ 0.05, *d* = 0.27). EE was similar during both halves (528 ± 58 vs. 514 ± 61 kcal; *p* = 0.60; *d* = 0.23), with 62% (second half: 65%) carbohydrate, 9% (9%) protein and 26% (27%) fat utilization. With respect to recovery, maximal voluntary knee extension (*p* ≥ 0.05, *d* = 0.50) and flexion force (*p* ≥ 0.05, *d* = 0.19), CMJ (*p* ≥ 0.05, *d* = 0.13), number of ground contacts (*p* ≥ 0.05, *d* = 0.57) and average contact time (*p* ≥ 0.05, *d* = 0.39) during 30-s of skipping remained unaltered 24 and 48 h after the game. Most ARSS dimensions of load (*p* < 0.05, *d* = 3.79) and recovery (*p* < 0.05, *d* = 3.22) returned to baseline levels after 24 h of recovery. Relative to baseline values, CK was elevated immediately and 24 h after (*p* < 0.05, *d* = 2.03) and normalized 48 h later.

**Conclusion:**

In youth soccer players the simulated match evoked considerable circulatory, metabolic and perceptual load, with an EE of 1042 ± 118 kcal. Among the indicators of perceived and performance fatigability examined, the level of CK and certain subjective ratings differed considerably immediately following or 24–48 h after a 2 × 40-min simulated soccer match in comparison to baseline. Accordingly, monitoring these variables may assist coaches in assessing a U17 player’s perceived and performance fatigability in connection with scheduling training following a soccer match.

## Introduction

In connection with their continuous efforts to improve performance, athletes are limited by the perceived and performance fatigability determined by their own physical and cognitive capacities (Enoka and Duchateau, 2016). The frequency, duration and intensity of training sessions, major determinants of performance (Halson, 2014), should be optimized with respect to the time-course of an athlete’s recovery from previous training/competition. For this purpose, the internal and external loads associated with various sports must be analyzed.

During a soccer match lasting 2 × 40 min, players under 17 years of age (U17) run 6000–8500 m, of which 52–84% is low- (<3 km⋅h^–1^) -to-moderate intensity exercise (3–8 km⋅h^–1^), 9–18% medium-to-high intensity (8–13 km⋅h^–1^) and 5–15% high-to-very-high intensity (>13 km⋅h^–1^) (Buchheit et al., 2010b; Castagna et al., 2010; Rebelo et al., 2014). Consequently, the internal load averages >80% of HR_peak_ (Mendez-Villanueva et al., 2013), with blood lactate values of 5.0 ± 2.3 mmol⋅l^–1^ (Aslan et al., 2012).

With respect to intra-match assessment of internal and external loads far less is known concerning youth compared to adult soccer players. In the latter, acute fatigue is related, at least in part, to the aerobic and anaerobic metabolic processes, such as depletion of glycogen stores (Mohr et al., 2003; Di Salvo et al., 2009), involved in elevating EE, with an average EE of 14.6 [as measured by video analysis (Osgnach et al., 2010)] to 16.8 and 18.1 kcal⋅kg^–1^ [determined by gas exchange (Bangsbo, 1994a; Ferrauti et al., 2006)]. Interestingly, to the best of our knowledge, corresponding characterization of a youth soccer match has not yet been reported and such information could assist with optimal energy intake during the match and breaks.

The weekly matches and competitive training of elite youth athletes involved in team sports cause neuromuscular fatigue with impairment of perceived recovery (McLean et al., 2010) and an elevated risk of fatigue-related injury (Aoki et al., 2012). Numerous investigations on adult soccer players have assessed the time-course of post-match or -exercise recovery employing various indicators of performance fatigability, including muscle damage (Byrne et al., 2004), maximal voluntary contraction force (Rampinini et al., 2011; Robineau et al., 2012; Nedelec et al., 2014; Brownstein et al., 2017; Goodall et al., 2017; Thomas et al., 2017), neuromuscular function (e.g., CMJ and LS20m) (Rampinini et al., 2011; Robineau et al., 2012; Nedelec et al., 2014; Brownstein et al., 2017; Thomas et al., 2017), and subjective fatigue (Robineau et al., 2012; Brownstein et al., 2017; Goodall et al., 2017; Thomas et al., 2017). Interestingly, far less is known about such factors in the case of youth soccer players than in adult athletes. Recently, U17 soccer players were reported to display more perceived fatigue and a slower 20-m sprint 24 h after a friendly match, with full recovery being attained after 48 h (Djaoui et al., 2016). The post-match kinetics of LS20m, subjective markers of RPE, blood lactate levels and muscle damage were also examined, but with no information on load during the match (Djaoui et al., 2016).

Both the magnitude of internal and external loads during the game and the time-course of recovery would appear to be of considerable practical relevance to youth athletes, who have a greater oxidative capacity and may therefore recover with respect to certain factors such as elimination of blood lactate and return of heart rate (HR) to baseline levels more rapidly than adults (Hebestreit et al., 1993; Falk and Dotan, 2006; Buchheit et al., 2010a; Engel et al., 2015). Such information would aid in determining (i) the energetic and nutritional strategies that best prevent premature performance fatigability during a match; (ii) the optimal timing of recovery and training; and (iii) the strategy for player rotation that prevents chronic fatigue and/or injury most effectively.

From a scientific perspective, a simulated soccer match [frequently utilized (Cone et al., 2012; Robineau et al., 2012; Goodall et al., 2017; Thomas et al., 2017)] offers numerous advantages over an actual match, including standardization of external loading (e.g., running intensity and profile), monitoring with sensors, comparison of individual players and less risk of injury or damage to the equipment.

The present investigation examined the internal intra-game load and time-course of recovery following completion of a simulated 2 × 40 min soccer match by elite youth soccer players. On the basis of previous investigations on simulated soccer games (Robineau et al., 2012; Rebelo et al., 2014; Brownstein et al., 2017; Goodall et al., 2017; Thomas et al., 2017), mainly involving adult athletes, we hypothesize that (i) the circulatory, metabolic and perceptual loads will be demanding throughout the simulation; (ii) perceived exertion will increase significantly with time; and (iii) test performance will be poorer after the match.

## Materials and Methods

### Participants

The 11 male youth players recruited from a local soccer club (age: 16 ± 1 years, body height: 178 ± 7 cm, body mass: 67 ± 7 kg) had all been competing for at least 6 years at the highest regional level for their age group, performing three training sessions and one competitive match each week.

The maturational status of the participants was assessed employing the Tanner stages based on pubic hair (Tanner and Whitehouse, 1976). Line drawings and written explanations of each developmental stage were utilized by the guardians for this staging, resulting in an average + SD of 4.4 ± 0.6.

All players and their legal guardians provided written consent to participate. This study was designed in accordance with the Declaration of Helsinki and approved by the institute’s ethical review board.

### Study Design

**FIGURE 1:**
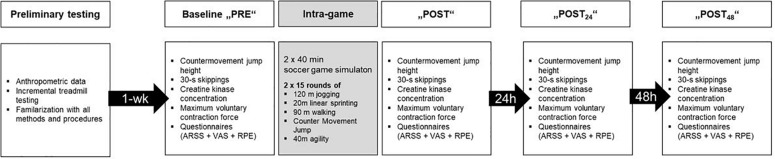
Timeline for all experiments, including preliminary testing, baseline (PRE) measurements, the soccer match simulation and measurements immediately (POST), 24 h (POST_24 h_) and 48 h later (POST_48 h_) after the match.

The overall study design is illustrated in Figure 1.

All participants visited the laboratory twice, first 1 week before the simulated match for anthropometric assessment, familiarization with the testing procedures and a ramp-like treadmill test to determine individual peak values of heart rate (HR_peak_) and oxygen uptake (VO_2__peak_) (Figure 1). The treadmill (H/P Cosmos, Mercury, Nussdorf-Traunstein, Germany) test was preceded by warm-up running at 10 km⋅h^–1^ (1% incline) for 5 min followed by a 4-min rest period. The test itself started at 10 km⋅h^–1^, with the velocity increasing 1 km⋅h^–1^ every 30 s thereafter, as described previously (Krustrup et al., 2006a).

#### Soccer-Match Simulation

**FIGURE 2:**
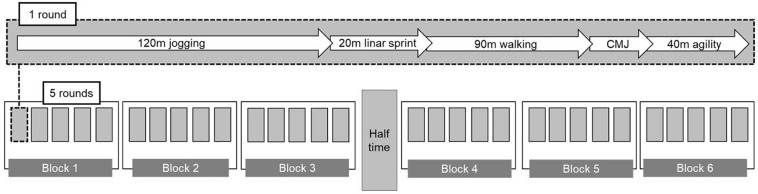
Schematic procedure of the soccer match simulation.

The simulated soccer match is illustrated schematically in Figure 2.

The simulated soccer match consisted of two 40-min sessions performed without a ball (to improve reproducibility), with a 10-min break between. Both halves included 15 rounds with different tasks performed in the following order: 120-m jogging, 20-m LS20m, 90-m walking, one CMJ and a 40-m agility assessment on a SC (Global Speed, Hemsbach, Germany). The total distance covered was 270 m per round and 8100 m for all 30 rounds. On the basis of previous findings on U17 youth soccer players (Buchheit et al., 2010b; Castagna et al., 2010; Mendez-Villanueva et al., 2013; Rebelo et al., 2014), the distances covered were calculated to involve approximately 45% jogging, 7% sprinting, 33% walking, and 15% running while performing a task requiring agility. All players were explicitly instructed to perform all sprints, jumps and agility trials at maximal effort.

#### Intra-Match Measurements

During the entire simulated soccer match, the participants wore a portable breath-by-breath gas analyzer for continuous monitoring of oxygen uptake, the respiratory exchange ratio, EE, substrate utilization, and HR. Blood samples for measurement of lactate were taken from the right ear lobe before the simulation, at the end of every five rounds and at the end of the 10-min break. In addition, the 20-m linear sprint time (LS20m), CMJ height (CMJ) and time required to perform an agility test were measured during each round. Perceived leg (RPE_leg_) and whole-body (RPE_total_) exertion were rated after every round.

During the 10-min half-time break, all players consumed 0.66 g⋅kg^–1^ of carbohydrates in the form of an isotonic drink (Iso-Sport, Sodenthaler, Sulzbach, Germany) and a gel (PowerGel, Active Nutrition International GmbH, Munich, Germany).

#### Pre- and Post-match Assessment of Perceived and Performance Fatigability

Several markers related to perceived and performance fatigability were evaluated prior to (PRE), immediately after (POST), as well as 24 (POST_24 h_) and 48 h (POST_48 h_) after completing the 2 × 40 min soccer match simulation. At each of these time-points, the CMJ height, the number of ground contacts and average contact time during 30-s of skipping, and the maximal voluntary isometric force of knee extension and flexion by each player were determined. In addition, capillary blood levels of CK, RPE_total_ and RPE_legs_, as well as scores on the ARSS and ratings of pain on a VAS were obtained. During the 48 h after the simulation, the players performed no further exercise.

### Procedures

#### Gas Exchange and Heart Rate

Oxygen uptake and exhalation of carbon dioxide were monitored continuously with an open-circuit breath-by-breath spirograph (MetaMax 3B, Cortex Biophysik, Leipzig, Germany), employing standard algorithms to compensate for the time delay between gas consumption and the signal. Both gas sensors were calibrated prior to each test with a precision gas mixture (15.8% O_2_ and 5% CO_2_ in N_2_; Praxair, Düsseldorf, Germany) covering the range of expected fractional gas concentrations. The volume sensor was calibrated with a precision 3-L syringe (Cortex Biophysik, Leipzig, Germany). The respiratory exchange ratio was calculated by dividing the volume of carbon dioxide exhaled (VCO_2_) by the oxygen consumption (VO_2_). HR was measured continuously by a chest belt (H7, Polar Electro Oy, Kempele, Finland) connected to the gas analyzer for temporal alignment with the respiratory data.

All respiratory values and HRs during ramp testing were averaged every 30-s, with the maximal oxygen uptake and HR being designated as VO_2__peak_ and HR_peak_, respectively. The percentages of VO_2__peak_ and HR_peak_ during the 2 × 40 m simulated match were employed as internal indicators of cardio-respiratory load.

#### Energy Expenditure and Substrate Analysis

Energy expenditure (kcal/min) was calculated on the basis of indirect calorimetry utilizing the formula 0.55 × VCO_2_ – 4.471 × VO_2_. The rates of fat and carbohydrate oxidation were calculated from VO_2_ and VCO_2_ using the following equations (Hetlelid et al., 2015):

Fatoxidation(g/min)=1.695×VO-21.701×VCO2

C⁢a⁢r⁢b⁢o⁢h⁢y⁢d⁢r⁢a⁢t⁢e⁢o⁢x⁢i⁢d⁢a⁢t⁢i⁢o⁢n⁢(g/m⁢i⁢n)=4.21×VCO-22.962×VO2

#### Blood Analysis

Capillary blood samples from the right earlobe were taken for the analysis of lactate (Lactate Pro 2, Arkray KDK, Kyoto, Japan) and CK (Reflotron Plus System, Roche, Basel, Switzerland) in duplicate, with the mean being utilized for statistical analysis. Upon repeated measurement, the standard errors for lactate and CK were 0.1 mmol⋅l^–1^ and 4.1 U⋅l^–1^, respectively.

#### Agility

Agility (i.e., repeated sprints with changes in direction in response to a visual stimulus) was examined with the recently developed SC (Global Speed, Hemsbach, Germany); this procedure and its reliability have been described in detail elsewhere (Duking et al., 2016). All changes-of-direction on the SC were pre-determined with five sprinting sequences of approximately 40 m each recurring six times in the same order unknown to the participants. The standard error of measurement was 0.3 s.

#### Linear Sprints

To examine the LS20m during each round, double light barrier timing gates (TC, Brower Timing Systems, Draper, UT, United States) were placed at the start and end of the 20-m sprinting path. The standard error of measurement was 0.2 s.

#### CMJ Height and 30-s Skipping

Countermovement jump height and the numbers of skips and contact time during 30 s were assessed with an optical measurement system (OptoJump, MicroGate, Bolzano, Italy). For assessment of the CMJ height during match simulation, the players were allowed to swing their arms actively to simulate soccer-specific movements. During PRE, POST, POST_24 h_ and POST_48 h_ evaluations, CMJs were performed with the hands on the hips. The standard error of measurement for the CMJ was 0.7. Skipping performance was assessed as the maximal number of skips during a 30-s period and the average contact time was also assessed. The standard errors of measurement for the number of skippings and contact time were 3.6 and 0.003 s, respectively.

#### Isometric Leg Force

Isometric leg force was examined during three 3-s maximal voluntary isometric contractions of the knee extensors and flexors employing a strength apparatus (EasyTorque, Tonus, Zemmer, Germany). This apparatus was positioned anatomically in accordance with the manufacturer’s recommendations and this position was the same in connection with the PRE, POST, POST_24 h_, and POST_48 h_ measurements. The standard errors of measurement for maximal voluntary isometric contractions of the knee extensors and flexors were 36 and 34.4 N, respectively.

#### Subjective Ratings

In connection with collection of LS20m times, CMJ height and agility performance during the 2 × 40 m match simulation, the participants were asked to rate their whole-body and leg exertion on Borg’s 6–20 scale (Borg, 1970).

Perceived leg pain was assessed employing a VAS ranging from no (0 cm) to unbearable pain (10 cm). The pain was classified as mild (0.1–3.8), moderate (3.9–5.7) or severe (5.8–10), as earlier (Boonstra et al., 2014).

In addition, the ARSS (Hitzschke et al., 2016), containing 32 adjectives grouped into eight scales, was used to assess individual levels of stress and recovery. A seven-point Likert-type scale ranging from 0 (does not apply) to 6 (applies fully) was employed for scaling. The stress-related scales include muscular stress, lack of activation, negative emotional state, and overall stress, while the recovery-related scales cover physical and mental performance, emotional balance, and overall recovery.

### Statistical Analysis

All data are presented as means ± standard deviations (SD) and CIs. Since all data were normally distributed as assessed by the Kolmogorov–Smirnov test, no transformation was required.

To compare intra-match cardio-respiratory, metabolic, perceptual and performance parameters over time, each 40-min half was split into three blocks (first half: B_1_–B_3_ and second half: B_4_–B_6_). One-way repeated-measures ANOVA was carried out for all variables at each time-point during the match (i.e., B_1_, B_2_, B_3_, B_4_, B_5_, B_6_), as well as to compare the value of each variable at pre- and post-testing (i.e., PRE, POST, POST_24 h_, POST_48 h_) to detect any differences between these blocks. Fisher *post hoc* analysis was applied to identify differences between the time-points, with an alpha of *p* < 0.05 being considered statistically significant. The effect size *d* according to Cohen (1988) was calculated for all values of B_1_ to B_6_ and at PRE, POST, POST_24 h_, and POST_48 h_, with 0.20, 0.50, and 0.80 being the thresholds for small, moderate and large effects. All statistical analyses were carried out with the Statistical software package for Windows^®^ (version 7.1, StatSoft Inc., Tulsa, OK, United States).

*A priori* power analysis employing the G^∗^Power software (Version 3.1.3, Heinrich-Heine University Duesseldorf, Germany) was used to determine an adequate sample size to test one-way repeated-measures ANOVA. Based on the effect sizes derived from previous studies analyzing the performance in CMJ before and after a simulated soccer match (Robineau et al., 2012; Thomas et al., 2017) as an expected main variable of performance fatigability, we calculated the sample sizes to range from *n* = 6–12 using an effect size *f* = 0.25, with a power coefficient of 0.8. Based on this analysis we expected a pre-defined sample size of 11 participants to be significant (*p* < 0.05) for all main variables.

## Results

### Intra-Match Variables

**FIGURE 3:**
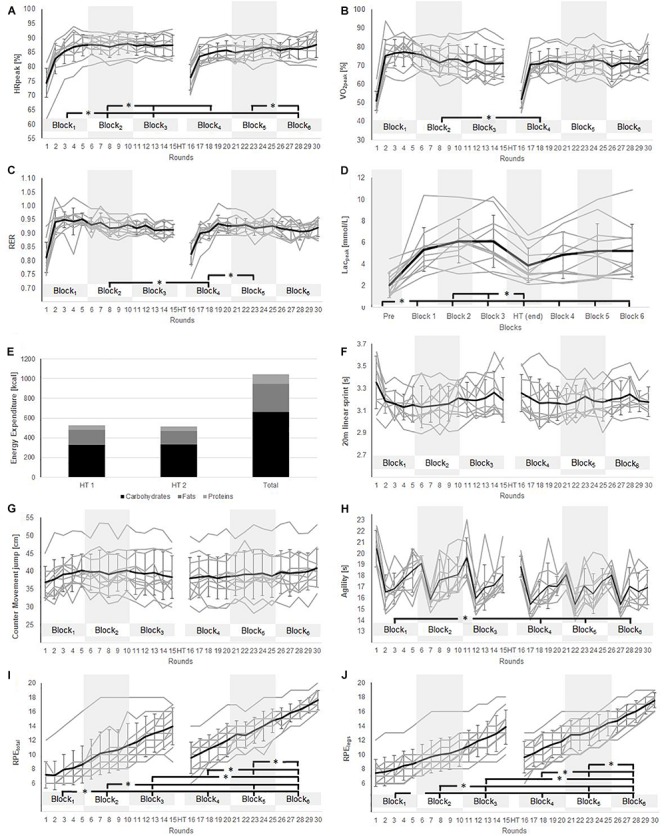
Inter-game time course and statistical analysis (^∗^*p* ≤ 0.05) of **(A)** %-heart rate peak; **(B)** %-peak oxygen uptake; **(C)** respiratory exchange ratio; **(D)** blood lactate concentration; **(E)** energy expenditure and substrate utilization; **(F)** 20 m linear sprint time; **(G)** counter movement jump height; **(H)** agility performance; **(I)** whole body perceived exertion, and **(J)** perceived exertion of the legs.

All intra-match variables are shown in Figure 3.

%HR_peak_ increased from B_1_ to B_2_ (83.4 ± 6.6% vs. 87.6 ± 3.3%; *p* < 0.05; *d* = 0.84) and from B_1_ to B_3_ (83.4 ± 6.6% vs. 87.4 ± 3.2%; *p* < 0.05; *d* = 0.82). HR_peak_ decreased during the half-time break (from 87.4 ± 3.2% at B3 to 83.1 ± 4.9% at B1; *p* < 0.05; *d* = 1.08) and rose from B_4_ to B_6_ (83.1 ± 4.9% vs. 86.5 ± 3.5%; *p* < 0.05; *d* = 0.81).

%VO_2__pea__k_ was higher at B_2_ than B_4_ (73.5 ± 7.6% vs. 67.6 ± 10.0%; *p* < 0.05; *d* = 0.68).

In comparison to baseline (2.0 ± 1.1 mmol⋅l^–1^), the levels of blood lactate were elevated at all other time-points [5.4 ± 2.0 (B_1_); 6.1 ± 2.0 (B_2_); 6.1 ± 2.4 (B_3_); 3.9 ± 1.6 (half-time); 4.9 ± 2.2 (B_4_); 5.2 ± 2.6 (B_5_); and 5.3 ± 2.5 (B_6_) mmol⋅l^–1^; all *p* < 0.05; *d* = 1.37–2.59].

The respiratory exchange ratio declined from B_2_ to B_4_ (0.93 ± 0.03 vs. 0.89 ± 0.05; *p* < 0.05; *d* = 0.74) and rose from B_4_ to B_5_ (0.89 ± 0.05 vs. 0.93 ± 0.03; *p* < 0.05; *d* = 0.70).

Energy expenditure was similar during both halves (528 ± 58 vs. 514 ± 61 kcal; *p* = 0.6, *d* = 0.23), as were the fractional substrate utilizations of carbohydrate (62% vs. 65%; *d* = 0.15), protein (9% vs. 9%; *p* = 0.16; *d* = 0.16) and fat (26% vs. 27%; *d* = 0.17).

Linear 20-m sprinting times were the same at all points of measurement [3.20 ± 0.17 (B_1_), 3.16 ± 0.16 (B_2_), 3.21 ± 0.17 (B_3_), 3.20 ± 0.18 (B_4_), 3.18 ± 0.14 (B_5_), 3.21 ± 0.14 (B_6_) s; *p* = 0.98; *d* = 0.01–0.34], as was the CMJ height [38.5 ± 5.3 (B_1_), 39.7 ± 5.8 (B_2_), 39.1 ± 6.2 (B_3_), 38.4 ± 6.1 (B_4_), 38.9 ± 6.0 (B_5_), 40.0 ± 5.7 (B_6_) cm; *p* = 0.97, *d* = 0.01–0.27].

Agility improved from B_1_ (18.0 ± 2.0 s) to B_4_ (16.9 ± 1.6 s; *p* < 0.05; *d* = 0.61), from B_1_ to B_5_ (16.9 ± 1.5 s; *p* < 0.05; *d* = 0.63) and from B_1_ to B_6_ (16.7 ± 1.5 s; *p* < 0.05; *d* = 0.70).

Rating of perceived exertion of the whole body increased gradually during the first half (7.8 ± 2.2 (B_1_) vs. 10.4 ± 2.9 (B_2_) vs. 13.0 ± 2.7 (B_3_); all *p* < 0.05; *d* = 0.94–1.01), as well as during the second half (10.8 ± 2.2 (B_4_) vs. 13.5 ± 2.0 (B_5_) vs. 16.3 ± 1.5 (B_6_); all *p* < 0.05; *d* = 0.9–1.61), as did RPE_legs_ from B_1_ to B_3_ (8.0 ± 2.0(B_1_) vs. 10.0 ± 2.2 (B_2_) vs. 12.4 ± 2.4 (B_3_); all *p* < 0.05; *d* = 0.92–1.07) and from B_4_ to B_6_ (10.8 ± 2.2(B_4_) vs. 13.4 ± 2.0 (B_5_) vs. 16.1 ± 1.7 (B_6_); all *p* < 0.05; *d* = 1.24–1.52).

### Indicators of Perceived and Performance Fatigability During and Recovery Following the Simulated Soccer Match

The findings on indicators of fatigability, recovery and performance before and following the simulated match are summarized in Tables 1, 2.

**TABLE 1:** Indicators of recovery from performance fatigability (Means ± SD) and 95% confidence intervals (95% CIs) before and immediately after (24 and 48 h) the simulated soccer match.

**Indicator**	**Time-point of measurement**					**Statistical analysis**	
	**PRE**	**POST**	**POST_24 h_**	**POST_48 h_**	**ES *d***	**P (main**	**P (*post hoc*)**
	**(±95% CIs)**	**(±95% CIs)**	**(±95% CIs)**	**(±95% CIs)**	**(min-max)**	**effects)**	**(min-max)**
Blood level of creatine kinase (U/l)	226 ± 134 (±79)	576 ± 211^a^ (±124.7)	762 ± 549^a^ (±324.7)	480 ± 356 (±210.5)	0.34–2.03	0.04	<0.05–0.52
Countermovement jump height (cm)	31.2 ± 4.2 (±2.49)	31.3 ± 4.2 (±2.49)	31.6 ± 5.0 (±2.98)	30.9 ± 4.9 (±2.91)	0.02–0.13	0.9	
Ground contacts during 30 s of skipping (*n*)	173.0 ± 23.1 (±13.7)	160.5 ± 26.5 (±15.7)	171.4 ± 21.9 (±12.9)	175.3 ± 25.8 (±15.3)	0.07–0.57	0.52	
Mean ground contact time during 30 s of skipping (s)	0.15 ± 0.03 (±0.015)	0.16 ± 0.03 (±0.021)	0.15 ± 0.03 (±0.016)	0.15 ± 0.02 (±0.013)	0.06–0.39	0.75	
**Maximal voluntary force**							
Knee extensors (*N*)	1140 ± 338 (±199.7)	1080 ± 332 (±196.0)	1250 ± 339 (±200.3)	1250 ± 365 (±216.0)	0.01–0.50	0.58	
Knee flexors (*N*)	531 ± 167 (±98.4)	518 ± 203 (±120.1)	499 ± 176 (±98.4)	504 ± 178 (±105.0)	0.03–0.19	0.97	

*ES = effect size; a = *p* < 0.05 vs. pre; b = *p* < 0.05 vs. post; c = *p* < 0.05 vs. 24 h; d = *p* < 0.05 vs. 48 h.*

**TABLE 2:** Subjective variables related to perceived fatigability (in arbitrary units) (Means ± SD) and 95% confidence intervals (95% CIs) before and immediately, 24 and 48 h after the simulated soccer match.

**Variable**	**Time-point of measurement**						**Statistical analysis**	
	**PRE**	**POST**	**POST_24 h_**	**POST_48 h_**	**ES *d***	**P (main**	**P (*post hoc*)**
	**(±95% CIs)**	**(±95% CIs)**	**(±95% CIs)**	**(±95% CIs)**	**(min-max)**	**effects)**	**(min-max)**
**Ratings of perceived exertion**							
Whole body	7.2 ± 2.1 (±1.26)	17.5 ± 1.3^a,c,d^ (±0.76)	8.6 ± 2.2 (±1.27)	7.3 ± 1.3 (±0.75)	0.05–6.04	0.03	<0.05–0.90
Legs	7.4 ± 1.9 (±1.13)	17.5 ± 1.0^a,c,d^ (±0.61)	10.6 ± 2.8^a^ (±1.68)	9.4 ± 2.7^a^ (±1.59)	0.46–6.91	0.04	<0.05–0.19
Physical performance	5.0 ± 0.4 (±0.26)	1.8 ± 1.4^a,c,d^ (±0.83)	4.0 ± 1.1^a,d^ (±0.65)	4.9 ± 0.5 (±0.32)	0.18–3.44	0.04	<0.05–0.82
Mental performance capability	5.2 ± 0.8 (±0.44)	2.2 ± 1.0^a,c,d^ (±0.58)	4.7 ± 1.2 (±0.70)	5.2 ± 0.6 (±0.36)	0.00–3.79	0.03	<0.05–1.00
Emotional balance	4.5 ± 1.2 (±0.72)	4.1 ± 1.1^c,d^ (±0.67)	5.0 ± 0.9 (±0.53)	5.0 ± 0.8 (±0.46)	0.00–0.95	0.03	<0.05–1.00
Overall recovery	4.9 ± 0.7 (±0.41)	1.7 ± 1.1^a,c,d^ (±0.65)	3.5 ± 1.6^a^ (±0.97)	4.4 ± 1.1 (±0.66)	0.59–3.53	0.04	<0.05–0.29
Muscular stress	0.5 ± 0.7 (±0.41)	4.1 ± 1.5^a,c,d^ (±0.89)	2.5 ± 1.6^a^ (±0.97)	1.8 ± 1.6^a^ (±0.95)	0.39–3.22	0.03	<0.05–0.30
Lack of activation	1.2 ± 1.4 (±0.83)	2.2 ± 1.7 (±1.02)	1.5 ± 1.6 (±0.97)	1.2 ± 0.8 (±0.44)	0.00–0.81	0.21	
Negative emotional state	1.4 ± 1.4 (±0.85)	1.3 ± 1.3 (±0.80)	0.9 ± 0.8 (±0.49)	1.0 ± 0.6 (±0.37)	0.07–0.40	0.43	
Overall stress	1.2 ± 1.1 (±0.64)	4.5 ± 1.0^a,c,d^ (±0.61)	2.4 ± 1.9 (±1.10)	1.5 ± 1.5 (±0.89)	0.21–3.18	0.04	<0.05–0.65
Visual analog scale	0.7 ± 1.0 (±0.58)	4.9 ± 3.7^a^ (±2.16)	3.6 ± 2.3^a^ (±1.37)	2.8 ± 2.5 (±1.50)	0.32–1.80	0.03	<0.05–0.65

*ES = effect size; a = *p* < 0.05 vs. pre; b = *p* < 0.05 vs. post; c = *p* < 0.05 vs. 24 h; d = *p* < 0.05 vs. 48 h.*

The blood levels of CK increased from PRE to POST_24 h_ (226 ± 134 vs. 576 ± 211 (POST) and 762 ± 550 (POST_24 h_) U⋅l^–1^; *p* < 0.05; *d* = 1.57–2.03) and returned to baseline-levels at POST_48 h_ [226 ± 134 (PRE) vs. 480 ± 356 U⋅l^–1^; *p* = 0.07; *d* = 1.04].

Countermovement jump height (*p* = 0.9; *d* = 0.02–0.13), total number of contacts (*p* = 0.52; *d* = 0.07–0.57) and average contact time (*p* = 0.75; *d* = 0.06–0.39) during the 30-s skipping test, as well as the maximal voluntary isometric force of the knee extensors (*p* = 0.58; *d* = 0.01–0.5) and knee flexors (*p* = 0.97; *d* = 0.03–0.19) did not differ between the time-points. Effect size calculation revealed the number of skippings during 30 s was lower immediately after the simulated match compared to PRE (*p* ≥ 0.05, *d* = 0.57) and maximal voluntary knee extension force was lower immediately after the simulation compared to POST_24 h_ (*p* ≥ 0.05, *d* = 0.50).

The RPE_total_ increased from PRE to POST (7.2 ± 2.1 vs. 17.5 ± 1.3; *p* < 0.05; *d* = 6.04) and declined again from POST to POST_24 h_ and POST_48 h_ (17.5 ± 1.3 vs. 8.6 ± 2.2 (POST_24 h_) and 7.3 ± 1.3 (POST_48 h_); all *p* < 0.05; *d* = 5.16–8.01).

The RPE_legs_ increased from PRE to POST (7.4 ± 1.9 vs. 17.5 ± 1.0; *p* < 0.05; *d* = 6.91) and decreased from POST to POST_24 h_ and POST_48 h_ (17.5 ± 1.0 vs. 10.6 ± 2.8 (POST_24 h_) and 9.4 ± 2.7 (POST_48 h_); all *p* < 0.05; *d* = 3.57–4.39).

All data concerning the ARSS and perception of pain are presented in detail in Table 1.

In contrast to emotional balance, subjective ratings of the other items related to recovery (physical and mental performance and overall recovery) declined from PRE to POST (*p* < 0.05; *d* = 3.44–3.53). All of these ratings, including those for emotional balance, were lower at POST than POST_24 h_ and POST_48 h_ (*p* < 0.05; *d* = 0.90–3.79). Furthermore, the assessment of physical performance was lower at POST_24 h_ than POST_48 h_ (4.0 ± 1.1 vs. 4.9 ± 0.5; *p* < 0.05; *d* = 1.11) and overall recovery even lower at POST_24 h_ than PRE (3.5 ± 1.6 vs. 4.9 ± 0.7; *p* < 0.05; *d* = 1.17).

Ratings of muscular and overall stress were higher at POST than PRE, POST_24 h_ or POST_48 h_ (*p* < 0.05; *d* = 1.04–3.22). Furthermore, muscular stress was perceived as greater at POST_24 h_ and POST_48 h_ than PRE (*p* < 0.05; *d* = 1.11–1.64).

Perception of pain was elevated at POST and POST_24 h_ in comparison to PRE (*p* < 0.05; *d* = 1.75–1.8).

## Discussion

Our aims here were (i) to assess the internal and external loads during a simulated soccer match involving elite youth players and (ii) to examine the intra- and post-match time-courses of recovery from perceived and performance fatigability.

The major findings concerning load were as follows:

1. The average oxygen uptake and HR were 71 ± 7% VO_2__peak_ and 86 ± 4% HR_peak_, respectively.
2. The level of blood lactate increased with time, reaching an average of 5.5 ± 2.3 mmol⋅l^–1^ by the end of the match simulation.
3. The total EE averaged 1040 ± 118 kcal.
4. Performance as assessed by LS20m, CMJ height and an agility test remained unaltered throughout both halves.
5. The ratings of perceived exertion increased continuously.

The major findings concerning recovery were the following:

1. The maximal voluntary isometric force of the knee flexors, LS20m CMJ height and average contact time during 30-s of skipping were the same at all time-points of measurement.
2. Analysis of variance analysis revealed no difference for the number of skippings performed during a 30-s period and the maximal voluntary isometric knee extension force at all time-points.
3. Blood levels of CK were elevated for as long as 24 h after the simulated match.
4. Most subjective feelings of recovery, stress and pain had returned to normal by POST_24 h_.
5. In comparison to PRE, RPE_total_ was elevated at POST only, whereas RPE_legs_ remained elevated for 48 h after the simulated match.

### Internal and External Loads During the Simulated Match

To quantify the internal load (in terms of HR, oxygen uptake, EE, and substrate utilization) during the soccer game, we required sophisticated equipment, including breath-by-breath analysis, as well as highly standardized pattern of movement. From a methodological perspective, only a match-simulation [as performed elsewhere (Cone et al., 2012; Robineau et al., 2012; Goodall et al., 2017; Thomas et al., 2017)] involving standardized running and walking distances, numbers of sprints, jumps and changes of direction could be used for our purposes. We pre-designed the running profile during our match on the basis of other analyses of U17 youth soccer matches (Buchheit et al., 2010b; Castagna et al., 2010; Mendez-Villanueva et al., 2013; Rebelo et al., 2014) and instructed each player to perform each sprint, jump and test of agility during B_1_–B_6_ with the greatest effort possible. Thus, we created a simulation whose demands closely resemble those of an actual soccer match.

In fact, our 2 × 40-min simulated soccer match resulted in an average 86% HR_peak_, a value similar to those obtained in analyses of actual youth soccer matches (Mendez-Villanueva et al., 2013; Rebelo et al., 2014). In addition, oxygen uptake averaged approximately 71% VO_2__peak_, in line with earlier indirect estimations (Bangsbo et al., 2006) based on VO_2_ measurements during a treadmill test (Bangsbo, 1994b; Krustrup and Bangsbo, 2001; Esposito et al., 2004). The similarity of our findings to those on real matches is strengthened further by the levels of blood lactate, with values here as high as 6 mmol/l, corresponding to earlier findings in adults (Ekblom, 1986; Krustrup et al., 2006b) and somewhat higher than that of 4.96 mmol⋅l^–1^ observed in youth soccer players of the same age (Aslan et al., 2012).

In sports involving intermittent loads, e.g., tennis and soccer (Ferrauti et al., 2001, 2006), indirect calorimetry has been employed to quantify relative substrate utilization, as well as EE. Our youth players expended 454 – 640 kcal (8.0 ± 0.8 kcal⋅kg^–1^, 62% carbohydrate, 29% fat, 9% protein) during the first and 427–623 kcal (7.7 ± 0.8 kcal⋅kg^–1^, 65% carbohydrate, 26% fat, 9% protein) during the second half of the match. Thus, these players appeared to benefit from consuming approximately 4.9 kcal/kg of carbohydrate during the HT break.

In comparison to an actual match, simulations may lead to different pacing strategies to maintain performance and prevent premature fatigue (Skorski and Abbiss, 2017). In an actual soccer match, depending on the player’s physique and duration of play, he/she regulates the distribution of EE by altering either exercise intensity and/or periods of recovery, especially under awareness of the effort of the ongoing task (Waldron and Highton, 2014). In this context the 15 20-m linear sprint times (see Figure 3) revealed U-shaped pacing during both halves. A similar pattern was observed for the agility test, where times tended to be shorter toward the end of the match-simulation. In this same context, players rated their perceived exertion as >13.5 toward the end of the first half and >17 at the end of the second half. Together, these findings indicate that the soccer players paced themselves to avoid premature fatigue before the end of the simulated match.

Interestingly, the cardiorespiratory and metabolic values and perceptional ratings differed during the course of the match. For example, after approximately 3–5 rounds of the simulation, circulatory and metabolic load reached a plateau; whereas RPE remained on average at <9. With time, oxygen uptake, HR and levels of blood lactate remained constant, while RPE rose continuously to 17.6 in the last round.

Furthermore, the CMJ jump height remained unaltered, even though RPE increased steadily. Since jumping is limited by neuro- muscular factors more than by aerobic and anaerobic energy consumption, in contrast to, e.g., intermittent sprinting, such maintenance of jumping performance seems entirely feasible. Indeed, adult soccer players showed no change in jumping performance following a competitive match (Robineau et al., 2012).

### Time-Course of Recovery From Perceived and Performance Fatigability

Several investigations (Robineau et al., 2012; Brownstein et al., 2017; Goodall et al., 2017; Thomas et al., 2017) on adult soccer players have analyzed the time-course of various variables related to neuromuscular, metabolic, cardiorespiratory and perceived fatigue to determine which of these variables is sufficiently sensitive and valid. Among these variables, blood levels of CK is one of the most frequently assessed indicators of muscle damage. Here, these levels were elevated immediately (POST) and 24 h (POST_24 h_) after the simulated match, with values averaging 576 ± 211 and 762 ± 549 U⋅l^–1^, respectively, values in line with previous observations on adults (Thomas et al., 2017). However, in contrast to findings on adult soccer players, where CK levels remained elevated (Thomas et al., 2017), these levels in our youth soccer players had returned to baseline 48 h after the simulation. Possible explanations for this difference include somewhat shorter exercise (80 min compared to 90 min in adults) and the assumptions that children perform high-intensity exercise with less power output than adults and recover more rapidly (Hebestreit et al., 1993; Falk and Dotan, 2006; Buchheit et al., 2010a; Engel et al., 2015).

As a simple non-invasive indicator, CMJ height has been utilized to assess neuro-muscular fatigue in adult soccer players (Robineau et al., 2012; Brownstein et al., 2017; Thomas et al., 2017). Only one report documented a significant decline in this height for as long as 72 h after a simulated soccer match (Thomas et al., 2017), while others report a decline for as long as 24 h (Brownstein et al., 2017) or no decline at all (Robineau et al., 2012) following simulated or competitive soccer play. Here, CMJ height did not differ between the time-points and we therefore conclude that this is not a sensitive indicator of fatigue in youth soccer players. In agreement, no changes in CMJ height were detected after simulated soccer match play by adults (Robineau et al., 2012). Although a CMJ involves a stretch-shortening cycle, a pattern of movement often prioritized when testing fatigue (Nicol et al., 2006), it may not necessarily reflect the overall decrement in production of force by a muscle (Wehbe et al., 2015). Previous findings have shown limited stretch-shortening during acceleration by youth soccer players (Chelly et al., 2010).

Several studies employing isometric leg force measurements to estimate neuromuscular fatigue have observed decreases following competitive (Brownstein et al., 2017) and simulated (Goodall et al., 2017) match play by adults. The maximal voluntary isometric force of the knee flexors of our U17 players showed no differences between the time-points of measurement and, therefore, like CMJ height, this might not be a sensitive indicator of neuro-muscular fatigue in youth soccer players, whereas effect size calculation revealed the maximal voluntary isometric force of the knee extensor to be somewhat lower in POST (1081 ± 332) compared to POST_24 h_ (1248 ± 339) and therefore might be considered as a possible indicator to detect performance fatigability. This assumption requires further investigations.

Also interestingly, and in contrast to the CMJ height and maximal voluntary isometric knee flexion force, the number of skippings during 30-s was indicated to be somewhat lower at PRE (*n* = 173 ± 23) than POST (*n* = 161 ± 27) by the associated effect sizes. Although this difference was not statistically significant, this parameter may provide a simple assessment of at least a certain dimension of fatigue in practice. Considering the high anaerobic load of 30-s of skippings, the sensitivity of this test may be explained by this demand of energy supply, which considerably exceeds the anaerobic alactacid area.

Almost all subjective ratings (i.e., ARSS, RPE_leg_, and RPE_total_) had returned to baseline 48 h after the 2 × 40-min match simulation. The two exceptions were the ratings of muscular stress and RPE_legs_, indicating more stress on the muscles of the lower body. Interestingly, the time-courses of these ratings differed from the objective assessment of muscle damage on the basis of blood levels of CK, which had returned to PRE values by POST_48 h_.

The time-course of recovery with respect to blood levels of CK, the number of skippings during 30 s, the maximal voluntary isometric knee extension force and certain aspects of subjective rating indicate that youth soccer players have recovered completely 24–48 h after a soccer game. However, LS20m, CMJ height and maximal voluntary isometric force of the knee flexors do not seem to be sufficiently sensitive to detect fatigue in youth soccer players following a 2 × 40 min game simulation.

### Methodological Considerations

Due to the training schedules and professional obligations of the players, we were not able to monitor all indicators of perceived and performance fatigability for 72 h, which, at least in adults, has been done before (Brownstein et al., 2017; Thomas et al., 2017). We are aware of the numerous measures of internal and external load, but after discussing several of these with the coach and guardians, decided on the ones used here.

In an actual match, numerous changes-of-direction in responses to a visual stimulus occur (Chaouachi et al., 2012), a physical and cognitive challenge that is difficult to stimulate. However, the SC, with its running paths unknown to the players, allows this to be done, at least to a certain degree.

Concerning the structure of the simulation, we did not analyze over-time load and to obtain maximal standardization, the patterns of movement were not related to different playing positions. Furthermore, since there were no teammates, opponents or interactions with the ball, technical and tactical aspects of play were lacking in the simulation.

### Limitations

All players regularly visit our laboratory for performance and health assessment. Within this assessment, all players perform linear and multidirectional sprints, different jump protocols and fill out questionnaires related to perceived exertion and recovery. In addition, during the familiarization trials, they all performed every test procedure at least twice to reduce potential learning effects. Thus, although with the study design chosen, we cannot definitively rule out the possibility that learning effects exerted an influence on the changes observed during and after the match, we are quite certain that our participants were well acquainted with all procedures. A randomized cross-over study might have eliminated this limitation, but the busy training schedules of our athletes made a control condition impossible.

## Conclusion

A simulated youth soccer match lasting 2 × 40 min involves considerable circulatory and metabolic internal loads, challenging both the aerobic and anaerobic systems. Neuromuscular performance did not decline, probably due to pacing. In light of the substrate depletion that occurs, in particular of carbohydrates, appropriate intake of nutrients is required to prevent impairment of energy-dependent performance.

Among the indicators of perceived and performance fatigability examined, the level of CK and certain subjective ratings differed considerably immediately following or 24–48 h after a 2 × 40-min simulated soccer match in comparison to baseline. Accordingly, monitoring these two variables may assist coaches in assessing a U17 player’s perceived and performance fatigability in connection with scheduling training following a soccer match.

## Ethics Statement

This study was designed in accordance with the Declaration of Helsinki and approved by the institute’s ethical review board.

## Author Contributions

BS, CZ, and PK designed the study. CZ and PK carried out the tests. BS and H-CH provided their expertise on how to analyze the data, carried out by PK. PK wrote the manuscript. All authors read and approved the manuscript.

## Conflict of Interest

The authors declare that the research was conducted in the absence of any commercial or financial relationships that could be construed as a potential conflict of interest.

## Abbreviations

ANOVA: analysis of variance
ARSS: acute recovery and stress scale
B_1_–B_6_: Block 1–6
CIs: 95% confidence intervals
CK: creatine kinase
CMJ: countermovement jump
EE: energy expenditure
HR_peak_: peak heart rate
HT: halftime
Lac_peak,_: peak lactate concentration
LS20m: linear sprinting (20 m)
RPE_leg_: rating of perceived exertion of the leg
RPE_total_: rating of perceived exertion of the whole body
SC: speedcourt
U17: under 17 years
VAS: visual analog scale
VCO_2_: carbon dioxide production
VO_2peak_: peak oxygen uptake.
